# Supplementary material for: WordCommentsAnalyzer: A windows software tool for qualitative research
Source: F1000Res. 2018 Sep 4;7:536. Originally published 2018 May 3. [Version 2] doi: 10.12688/f1000research.14819.2 (PMC6137411; doi:10.12688/f1000research.14819.2)
Supplement: Supplementary file 3 [file f1000research-7-17614-s0002.tgz › b2a79496-4ada-47c4-9682-a99ce867836d.zip!/Eight Iranian Health Journals/Journal of Research in Rehabilitation Sciences _42.docx]

© Journal of Research in Rehabilitation Sciences . This work is licensed under CC BY-NC 4.0 (https://creativecommons.org/licenses/by-nc/4.0/).

Volume & Issue: دوره 7، شماره 4: 1390

Title: بررسی ارتباط بین قدرت Pinch و Grip با سرعت هدایت عصبی مدین

http://jrrs.mui.ac.ir/index.php/jrrs/article/view/434

مقدمه: ساختار دست انسان از سازمان‌دهی پیچیده‌ای برخوردار بوده است که به دنبال هر گونه ضایعه، هر چند خفيف دچار کاهش قدرت و کارایی خود می‌گردد. اندازه‌گیری قدرت Pinch و Grip جهت تهیه یک شاخص كمي از صحت عملکرد دست توسط داینامومتر به عنوان دقیق‌ترین و قابل قبول‌ترین روش پذیرفته شده است. سرعت هدایت عصبی (Nerve conduction velocity یا NCV) روش کلینیکی غیر تهاجمی دیگری است که برای محاسبه سرعت هدایت و سلامت عصب استفاده می‌شود. مواد و روش‌ها: این مطالعه از نوع مقطعی بود که بر روی ۳۹ زن که شرايط ورود به مطالعه را داشتند، پس از ارزيابي NCV اندام فوقاني، انجام گرفت. قدرت انواع Pinch و Grip هر دو دست اين افراد پس از تكميل فرم رضایت‌نامه تحقیق و پرسش‌نامه مربوط به ارزيابي عصب مدين، با داينامومتر اندازه‌گيري گردید. سپس اطلاعات به دست آمده با يافته‌هاي الكترودياگنوستيك هر نمونه با نرم‌افزار SPSS و آزمون ضریب همبستگی Pearson مورد تجزیه و تحلیل قرار گرفت. یافته‌ها: بين سرعت هدایت عصبی، حسي و حرکتی عصب مدين با انواع Pinch و Grip رابطه معني‌دار مثبتي وجود داشت (بیش‌ترین رابطه بین سرعت هدایت عصبی- حرکتی با 1Palmar pinch، 001/0 > P، 379/0 = r و بیش‌ترین رابطه Grip با سرعت هدایت عصبی- حرکتی 002/0 > P، 342/0 = r). بين شاخص ميزان درگيري عصب مدين، انواع Pinch و Grip رابطه معني‌دار منفي وجود داشت (بیش‌ترین رابطه با 1Palmar Pinch، 001/0 > P، 613/0- = r و Grip، 001/0 > P، 563/0- = r). بين شاخص ميزان درگيري عصب مدين و سرعت هدایت عصبی، حسي و حركتي آن رابطه معني‌دار منفي وجود داشت (بیش‌ترین رابطه با سرعت هدایت عصبی- حسي 001/0 > P، 562/0- = r).  نتیجه‎گیری: اندازه‌گيري قدرت عضلات ناحیه تنار در ارزيابي سرعت هدايت عصبي مدين كاربرد محدودی دارد. پرسش‌نامه شاخص ميزان درگيري عصب مدين نيز به عنوان ابزار باليني استاندارد در پيش‌بيني سرعت هدایت عصبی، حسي و حركتي مدين قابل توجه است.

Title: بررسي بسامد پايه در تمايزگذاري صوت طبيعي و مبتلا به ندول

http://jrrs.mui.ac.ir/index.php/jrrs/article/view/227

مقدمه: ندول صوتی، یکی از ضایعات تارهای صوتی است. ندول‌های صوتی به عنوان نتیجه عادات غلط تار صوتی و استفاده نادرست از صدا به شمار می‌آیند. ندول واکنش بافتی به فشار پایدار، شامل حرکات مکرر و شدید است. نشانه‌های آکوستیکی افراد با ندول تارهای صوتی تا حدودی با افراد دارای صوت طبیعی متفاوت است. اما در مورد اندازه‌های آکوستیکی متفاوت گزارش شده توافق نظری وجود ندارد. در پژوهش حاضر، از متن استاندارد رنگین کمان برای ارزیابی صوت افراد طبیعی و افراد مبتلا به ندول صوتی استفاده شد. مواد و روش‌ها: پژوهش حاضر از نوع توصیفی- تحلیلی بود، که به صورت مقطعی انجام شد. در این پژوهش افراد از گروه‌های سنی 40 تا 59 ساله انتخاب شدند که در 2 گروه سنی 49-40 و 59-50 ساله مورد ارزیابی قرار گرفتند. از هر گروه سنی 5 زن و 5 مرد با صوت طبیعی و 5 زن و 5 مرد مبتلا به ندول صوتی انتخاب گردید. بنابراین در هر گروه سنی 20 نفر بررسی شدند. روش انتخاب آزمودنی‌ها تصادفی بود. متغیرهای مورد بررسی شامل میانگین و دامنه تغییرات بسامد پایه بود. این پارامترها در افراد مبتلا به ندول دو طرفه تارهای صوتی و افراد طبیعی مقایسه شد. یافته‌ها: تفاوت میانگین بسامد پایه صوت در مجموع نمونه‌ها معنی‌دار بود (043/0 = P). برای تعیین محل تفاوت، آزمون ANOVA در زنان و مردان تکرار شد. در مردان تفاوت معنی‌داری بین میانگین بسامد پایه در دو گروه و در گروه‌های سنی مشاهده نگردید (59/0 = P). در زنان تفاوت میانگین بسامد پایه در دو گروه معنی‌دار بود (007/0 = P). نتیجه‎گیری: آنالیز میانگین بسامد پایه فقط در گروه سنی 59-50 ساله، می‌تواند بین صوت طبیعی و صوت مبتلا به ندول تمایزگذاری کند. با وجود مشخص بودن ندول در استروبوسکوپی بسته به میزان تأثیر بر صدا، خصوصیات آکوستیکی متفاوت خواهند بود.

Title: تأثیر برنامه پیش‌گیری کننده آسیب هارمونی بر قدرت ایزومتریک عضلات مفصل زانو در فوتباليست‌هاي مرد حرفه‌ای جوان

http://jrrs.mui.ac.ir/index.php/jrrs/article/view/262

مقدمه: کمبود قدرت عضلانی و یا عدم تعادل عضلانی، یکی از ریسک فاکتورهای داخلی آسیب می‌باشد. انجام دادن مهارت‌هاي تکنيکي و تاکتيکي و کاهش آسيب در فوتبالیست‌ها، به وجود قدرت کافی در عضلات بستگی دارد. هدف تحقیق حاضر بررسی تأثیر برنامه پیش‌گیری کننده آسیب هارمونی بر قدرت ایزومتریک عضلات مفصل زانو در فوتباليست‌هاي مرد جوان حرفه‌اي ایران بود. مواد و روش‌ها: 24 فوتبالیست حرفه‌ای زیر 21 سال، که دارای سابقه ورزشی حداقل 5 سال بوده‌اند، به صورت هدفمند به عنوان نمونه آماری در دو گروه تجربی (12 نفر) و شاهد (12 نفر) انتخاب شدند. در این تحقیق از برنامه تمرینی از قبل طراحی شده پیش‌گیری از آسیب‌های زانو به نام هارمونی استفاده شد. این برنامه تمرینی به مدت 8 هفته و هر هفته 3 بار در محدوده زمانی بین ساعت 8-4 عصر انجام شد. اندازه‌گيري قدرت ايزومتريک عضلات زانو در 3 زاويه 30، 60 و 90 درجه به وسیله دستگاه ایزوکنیتیک بایودکس انجام شد. ارزیابی‌های آماری به وسیله آزمون‌های Dependent t-test ، Independent t-test و آنالیز مکرر دو طرفه (Two way repeated measure) انجام شد. یافته‌ها: قدرت ایزومتریک عضلات چهارسر گروه تجربی در زاویه 60 و 90 درجه در آزمون نهایی بیشتر از پیش‌آزمون مشاهده شد و این مقدار از لحاظ آماری معنی‌دار بود (05/0 < P). همچنین تفاوت معنی‌داری در قدرت عضلات همسترینگ در آزمون نهایی نسبت به پیش‌آزمون در زوایای 30 و 60 درجه وجود داشت (05/0P <). نتایج آنالیز مکرر دو طرفه نشان داد که قدرت عضلات چهارسر گروه تجربی در زوایای مختلف تفاوت معنی‌داری با یکدیگر داشتند (001/0 < P، 81 = 11، 1F)؛ به طوری که بیشترین مقدار قدرت در زاویه 90 درجه (1/292 نیوتن متر) مشاهده شد. تفاوت معنی‌داری در قدرت عضلات همسترینگ گروه تجربی در هر سه زاویه با یکدیگر وجود داشت (001/0 < P، 5/122 = 11، 1F)؛ در حالی که بیشترین مقدار قدرت تولید شده به وسیله عضلات همسترینگ در زاویه 30 درجه (1/155 نیوتن متر) بود. نتیجه گیری: برنامه تمرینی هارمونی موجب افزایش قدرت عضلات چهارسر و همسترینگ فوتبالیست‌های مرد حرفه‌ای شده است. جایگزین کردن برنامه تمرینی هارمونی با روش گرم کردن سنتی فوتبال، در پیش‌گیری از آسیب‌های پایین تنه به دلیل افزایش قدرت عضلات زانو، می‌‌تواند مفید و مؤثر باشد.

Title: بررسي رابطه وضعيت شنوايي با درك واژه‌هاي مترادف در دانش‌آموزان پايه پنجم ابتدايي

http://jrrs.mui.ac.ir/index.php/jrrs/article/view/225

مقدمه: کودکان آسیب‌دیده شنوایی شدید تا عمیق در درک و اکتساب زبان گفتاری مشکلات زیادی دارند. امروزه تحقیقات از اندازه‌گیری میزان واژگان به سمت درک فرایندهای یادگیری واژه‌ها، که شامل رشد مقوله معنی‌شناسی در این کودکان است، سوق داده شده‌اند. هدف از اين پژوهش، بررسي تفاوت بين دانش‌آموزان شنوا و آسیب‌‌دیده شنوایی، در یکی از جنبه‌های معنی‌شناسی و درك واژه‌هاي مترادف بوده است. مواد و روش‌ها: اين مطالعه از نوع توصيفي– تحليلي- مقطعي بود. در اين پژوهش 78 دانش‌آموز پايه پنجم ابتدايي شهر اصفهان در دو گروه 52 نفري شنوا و 26 نفری آسيب‌ديده شنوايي مورد مطالعه قرار گرفتند. در جمع‌آوري داده‌ها از دو آزمون نوشتاري استفاده شد؛ كه آزمودني‌ها مي‌بايست در يك آزمون، مترادف واژه مورد نظر را در بافت جمله و در آزمون ديگر، مترادف واژه را در خارج از بافت جمله مي‌نوشتند. یافته‌ها: تفاوت معنی‌داری بین گروه شنوا و گروه آسیب شنوایی در هر دو آزمون مشاهده گردید (05/0 > P). توانايي هر دو گروه دانش‌آموز در درك واژه‌هاي مترادف در بافت جمله، به طور معنی‌داري بالاتر از درك واژه‌هاي مترادف در خارج از بافت جمله بود (05/0 > P). نتیجه‎گیری: با توجه به اختلاف بين گروه شنوا با گروه آسيب‌ديده شنوايي، به نظر مي‌رسد كه مقوله معنی‌شناختي همچون ساير مهارت‌هاي زباني تحت تأثير سلامت شنوايي مي‌باشد. به علاوه تفاوت نمرات در دو آزمون، نشان دهنده ساده‌تر بودن آزمون درک واژه‌های مترادف در بافت جمله مي‌باشد.

Title: بررسي مقايسه‌اي شيوع عوامل خطر مرتبط با كمردرد در زنان شهري و روستايي 18 تا 60 ساله شيراز و روستاهاي اطراف آن

http://jrrs.mui.ac.ir/index.php/jrrs/article/view/172

مقدمه: تخمين زده مي‌شود که حدود 84 درصد انسان‌ها در طول زندگي خود كمردرد را تجربه مي‌كنند. با توجه به نقش كليدي زنان به خصوص زنان روستايي در مشاركت‌هاي اقتصادي، خانوادگي و حتي در سطح ملي بررسي عوامل و فاكتورهاي ايجاد كمردرد در زنان مي‌تواند گامي مؤثر در امر پيش‌گيري و افزايش سلامت عمومي جامعه باشد. لذا اين مطالعه با هدف مقايسه و شناخت عوامل خطرزا و ايجاد كننده كمردرد در دو قشر زنان ساكن روستا و شهر طراحي شده است. مواد و روش‌ها: جامعه مورد مطالعه زنان شهري و روستايي 18 تا 60 ساله مبتلا به كمردرد ساکن در شيراز و روستاهاي اطراف بودند كه به روش نمونه‌گيري آسان انتخاب شدند. پرسش‌نامه محقق‌ساخته با Cronbach's alpha 70 درصد، ميان 646 زن در هر دو گروه توزيع شد و سپس اطلاعات جمع‌آوري شده در نرم‌افزار 16SPSS بررسي گرديد. یافته‌ها: متغيرهايي نظير سن، شاخص توده بدني، تعداد فرزندان، تعداد دفعات حاملگي، ساييدگي مفاصل و آلرژي، وضعيت غالب ستون فقرات هنگام كار، نحوه خوابيدن، زمان انجام بخش اعظم كار، استراحت بين ساعات كاري و گزارش درد در ستون فقرات طي 6 ماه گذشته در دو گروه شهري و روستايي اختلاف آماري معني‌داري نداشتند. در حالي كه تفاوت آماري معني‌داري از نظر وضعيت تأهل و تحصيل، فعاليت ورزشي منظم، نحوه جابجايي اجسام سنگين، نوع رختخواب، مدت انجام كار بيش از 8 ساعت طي 6 ماه گذشته، كل مدت استراحت در طول فعاليت و تعداد دفعات ناراحتي ستون فقرات طي 6 ماه گذشته بين دو گروه مشاهده شد (0001/0 = P). نتیجه گیری: با وجود شباهت‌هاي زياد در ويژگي‌هاي دموگرافيك زنان روستايي و شهري شركت كننده در اين مطالعه، تفاوت‌هايي نظير عدم استفاده از تشك طبي، مصرف بيشتر دخانيات و قليان، فقدان برنامه ورزشي منظم و كافي در طول زندگي، ساعات بيشتر سر پا ايستادن و سطح تحصيلات پايين در اکثر گزارشات ناراحتي ستون فقرات زنان روستايي اثرگذار بوده است.

Title: تعیین اعتبار درون و بین ارزیاب "آزمون ارزیابی گفتار افراد مبتلا به شکاف کام بر اساس سیستم پارامترهای جهانی- نسخه فارسی"

http://jrrs.mui.ac.ir/index.php/jrrs/article/view/417

مقدمه: شکاف لب و کام یکی از شایع‌ترین نقایص مادرزادی است. افراد مبتلا به شکاف کام ویژگی‌های گفتاری خاصی دارند که با ارزیابی ادراکی گفتار، توسط آسیب شناسان گفتار و زبان، شناسایی می‌شوند. ارزیابی ادراکی، استانداردی طلایی برای ارزیابی گفتار است. بر اساس این ویژگی‌های گفتاری و شیوع شکاف کام، لازم است از آزمونی پایا، برای ارزیابی ادراکی خطاهای گفتاری افراد مبتلا به شکاف کام استفاده نمود. مواد و روش‌ها: در این مطالعه توصیفی- تحلیلی و مقطعی، به منظور تعیین اعتبار درون و بین ارزیاب "آزمون ارزیابی گفتار افراد مبتلا به شکاف کام بر اساس سیستم پارامترهای جهانی- نسخه فارسی"، نمونه گفتاری 34 کودک 5 تا 9 ساله مبتلا به شکاف کام، مراجعه کننده به کلینیک شکاف کام اصفهان، توسط دو آسیب شناس گفتار و زبان (کم‌تجربه و باتجربه)، مورد ارزیابی قرار گرفتند. 30 درصد از نمونه‌ها به طور تصادفی انتخاب و بعد از دو ماه، توسط همان دو ارزیاب ارزیابی شدند. مواد گفتاری شامل 40 تک واژه و 17 جمله کنترل شده و گفتار پیوسته بود. میزان اعتبار درون ارزیاب با استفاده از ضریب همبستگی درون طبقه‌ای (intraclass correlation coefficient یا ICC) و میزان اعتبار بین ارزیاب با استفاده از ضریب همبستگی کندال و ICC برآورد گردید. یافته‌ها: میانگین میزان اعتبار درون ارزیاب با استفاده از ICC، برای ارزیاب کم تجربه 967/0 و برای ارزیاب باتجربه 971/0، برآورد گردید. میانگین میزان اعتبار بین ارزیاب با استفاده از ضریب همبستگی کندال، 953/0 و با استفاده از ICC، 957/0 برآورد گردید. نتیجه گیری: این ابزار از اعتبار درون و بین ارزیاب قابل قبولی برای ارزیابی گفتار افراد مبتلا به شکاف کام برخوردار بود. از این ابزار می‌توان به عنوان ابزاری پایا در مطالعات مربوط به گفتار کودکان 5 تا 9 ساله مبتلا به شکاف کام، استفاده نمود.

Title: مقایسه عملکرد ارتز MTK-RGO با ارتزهای رایج برای ایستادن افراد آسیب نخاعی

http://jrrs.mui.ac.ir/index.php/jrrs/article/view/266

مقدمه: افراد مبتلا به آسیب‌های نخاعی بیشتر از ویلچر برای جابجایی از مکانی به مکان دیگر استفاده می‌کنند، اما این افراد برای بهبود وضعیت سلامتی خود باید با استفاده از ارتز بایستند و راه بروند. اگر چه ارتزهای متعددی برای افراد دارای آسیب نخاعی طراحی شده است، این افراد با مشکلات مختلفی به هنگام استفاده از این ارتزها روبرو هستند. یک نوع جدید از ارتزهای رسیپروکال (Reciprocal gait orthosis) در بخش مهندسی پزشکی دانشگاه استرتکلاید (Strathclyde university) در جهت حل مشکلات ارتزهای موجود طراحی شده است. از آن جا که تاکنون تحقیقی برای بررسی عملکرد این ارتز بر روی افراد آسیب نخاعی انجام نشده است، تحقیق حاضر به ارزیابی عملکرد ارتز جدید برای ایستادن افراد آسیب نخاعی پرداخته است. مواد و روش‌ها: در این مطالعه که از نوع کارآزمایی بالینی بود، 5 فرد مبتلا به آسیب نخاعی که سطوح آسیب دیدگی آن‌ها بین 4T تا 12T بود، مورد بررسی قرارگرفتند. علاوه بر این یک گروه کنترل از افراد سالم که از لحاظ سن و قد با گروه آسیب نخاعی مشابه بودند، نیز در تحقیق شرکت کردند. پایداری افراد در هنگام ایستادن و همچنین هنگامی که با استفاده از دست فعالیت‌هایی را انجام می‌دادند، در دو حالت یکی با ارتز جدید و دیگری با ارتزهای KAFO (Knee ankle foot orthosis) ارزیابی شده است. تفاوت میان عملکرد افراد آسیب نخاعی هنگامی که از دو نوع ارتز استفاده کردند و همچنین تفاوت میان عملکرد افراد سالم و افرادآسیب نخاعی به ترتیب با استفاده از Paired t-test و Two-sample t-test مقایسه گردید. به منظور تجزیه و تحلیل داده‌ها از 16SPSS و سطح معنی‌داری 05/0 > P بهره‌گیری شد. یافته‌ها: پایداری افراد آسیب نخاعی به هنگام ایستادن با ارتز جدید از ارتزهای KAFO بهتر ارزیابی گردید (05/0 > P). علاوه بر این نیروی وارده بر عصا در استفاده از دو نوع ارتز، مقادیر متفاوتی را نشان داد (013/0 = P). عملکرد افراد آسیب نخاعی به هنگام انجام فعالیت‌های دست با ارتز جدید بهتر بود (05/0 = P). نتیجه گیری: توانایی برای تغییر راستای اجزای ارتز جدید نسبت به یکدیگر، سختی بالای این ارتز و تناسب بهتر ارتز با فرد دلایل اصلی عملکرد بهتر ارتزهای موجود می‌باشد.

Title: بررسی نیازهای محیطی بیماران و معلولین 6 تا 12 سال بر پایه تجارب آن‌ها در جهت کیفیت بخشیدن به فضاهای درمانی کودکان در شهر اصفهان: یک مطالعه پدیدارشناسی

http://jrrs.mui.ac.ir/index.php/jrrs/article/view/376

مقدمه: از آن جا که بررسی تجارب و نیازهای محیطی کودکان بیمار و معلول در محیط‌های درمانی، می‌تواند در ارتقای کیفیت این فضاها و در نهایت ایجاد آرامش بیشتر در هنگام درمان مؤثر باشد، نتایج این تحقیق می‌تواند در جهت ایجاد محیط درمانی مطلوب برای کودکان، کمک نماید. مواد و روش‌ها: این پژوهش به روش کیفی از نوع پدیدارشناسی و بر اساس نمونه‌گیری هدفمند ملاک محور بوده است. بنابراین، از بین کودکان بیمار و همچنین معلول مبتلا به Head injury، Spina bifida، Spinal cord injury و انواع دفورمیتی اندام تحتانی، 35 با گروه سنی 6 تا 12 سال مورد بررسی قرار گرفتند. از کودکان درخواست شد بر اساس تجاربشان، فضای درمانی مطلوب و نیازهای خود را توسط نقاشی به تصویر بکشند. علاوه بر این، پژوهش‌گر با استفاده از مکالمه نیمه‌ساختار یافته باز عمیق، به کودک در تکمیل نقاشی خود و بیان نیازها، کمک می‌نمود. برای تجزیه و تحلیل تصاویر و متن‌های به دست آمده، از روش تحلیل روایت و از کدگذاری موضوعی توسط نرم‌افزار Atlas Ti6 استفاده گردید. یافته‌ها: از تصاویری که توسط کودکان کشیده شد، 5 موضوع اصلی استخراج گردید که شامل تفریحات و عوامل سرگرم کننده، مبلمان، زیبایی و تنوع محیطی، ارتباطات احساسی- اجتماعی و حس امنیت بود. نتیجه گیری: یافته‌ها نشان داد که استفاده از تجارب کودکان در کیفیت بخشیدن به محیط‌های درمانی، از اهمیت زیادی برخوردار است و می‌توان با استفاده از نیازهای مطرح شده توسط آن‌ها که شامل طبیعت، تزیینات، رنگ‌های شاد و مطلوب و نور طبیعی و مناسب بود، محیطی مطلوب را در فضاهای درمانی ایجاد نمود که کودک در حین درمان احساس آرامش داشته، در نهایت در تسریع بازتوانی کودک مؤثر باشد.

Title: بررسی تأثير ترکیب تکنیک‌های واقعیت مجازی و حرکت درمانی همراه با محدودیت بر عملکرد اندام فوقانی كودكان مبتلا به فلج مغزي همي‌پارزی

http://jrrs.mui.ac.ir/index.php/jrrs/article/view/178

مقدمه: فلج مغزی همی‌پارزی، آسیب بیشتر یک سمت بدن در نتیجه اختلالی غیر پیش‌رونده در مغز نابالغ است. هدف این مطالعه تعیین تأثیر ترکیب دو روش درمانی واقعیت مجازی و حرکت‌درمانی همراه با محدودیت بر عملکرد اندام فوقانی کودکان مبتلا به فلج مغزی همی‌پارزی بود. مواد و روش‌ها: تعداد 32 کودک فلج مغزی همی‌پارزی از جامعه در دسترس شهر اهواز در یک مطالعه کارآزمایی بالینی تصادفی یک سویه کور، در 4 گروه 8 نفره بررسی شدند (گروه واقعیت مجازی، گروه حرکت‌درمانی همراه با محدودیت، گروه ترکیبی و گروه شاهد). برنامه درمانی شامل جلسات 5/1 ساعته، یک روز در میان و به مدت 4 هفته بود. ارزیابی توسط ابزار کفایت حرکتی برواینینکس- اوزرتسکی و فعالیت حرکتی کودکان، قبل، بعد و 3 ماه پس از اتمام مداخلات صورت گرفت. از نرم افزار 16SPSS جهت انتخاب تصادفی نمونه‌ها و همچنین تحلیل داده‌ها با استفاده از آزمون آنالیز واریانس در تکرار مشاهدات در سطح معنی‌داری 05/0 استفاده گردید. یافته‌ها: آنالیز اطلاعات ابزارهای ارزیابی حاکی از اختلاف معنی‌دار آماری در گروه ترکیبی نسبت به گروه‌های مجزای درمانی و شاهد بود (01/0 P <) (ابزار کفایت حرکتی Bruininks–Oseretsky: تغییر میانگین سرعت و مهارت از 08/0 ± 15/0 به 33/0 ± 89/1 در جلسه پس از درمان بود). آنالیز اطلاعات جلسه پی‌گیری ارزیابی نیز حاکی از عدم وجود تفاوت معنی‌دار آماری بین جلسات پس از درمان و پی‌گیری در هر 4 گروه مطالعه بود (32/0 = P).  نتیجه گیری: تلفیق 2 روش درمانی واقعیت مجازی و حرکت‌درمانی همراه با محدودیت به دلیل کسب مزایای مثبت هر دو روش و اجرای آن‌ها در قالب یک برنامه درمانی، می‌تواند سبب بهبودی بیشتری در عملکرد اندام فوقانی کودکان مبتلا به فلج مغزی همی‌پارزی گردد.

Title: بررسی تأثیر تمرین مقاومتی نشستن به ایستادن بر عملکرد حرکتی درشت در کودکان فلج مغزی دایپلژی اسپاستیک

http://jrrs.mui.ac.ir/index.php/jrrs/article/view/328

مقدمه: یکی از عوامل مهم اختلالات حرکتی در فلج مغزی دایپلژی اسپاستیک، محدودیت عملکردهای حرکتی درشت به علت ضعف عضله است. با توجه به تأثیر قدرت بر عملکرد حرکتی درشت، هدف از این تحقیق، بررسی تأثیر تمرین مقاومتی نشستن به ایستادن بر عملکرد حرکتی درشت در کودکان فلج مغزی دایپلژی اسپاستیک بود. مواد و روش‌ها: این مطالعه بر روی 20 کودک فلج مغزی دایپلژی اسپاستیک 5 تا 12 ساله، شامل 12 پسر و 8 دختر با میانگین سنی 30/8 ± 89/1 انجام شد. آزمودنی‌ها طي 6 هفته و هر هفته 3 جلسه تمرین مقاومتی نشستن به ایستادن با وزنه را انجام دادند. قدرت ایزومتریک عضلات اکستانسور ران، اکستانسور زانو و پلانتار فلکسور مچ پا توسط دستگاه Nicholas manual muscle testing (Nicholas MMT) ارزیابی شد. در ضمن عملکرد حرکتی درشت توسط ابزار Gross motor function measure (GMFM) ارزیابی شد. داده‌های حاصل با آزمون  Paired t-testمورد تجزیه و تحلیل قرار گرفت. یافته‌ها: قدرت گروه‌های عضلانی اکستانسور ران و اکستانسور زانو به صورت معنی‌داری (0001/0 > P) بعد از مداخله بهبود یافت، در حالی که در قدرت گروه عضلانی پلانتار فلکسور مچ پا تغییری ایجاد نشد (573/0 ₌ P). همچنین در آزمون GMFM در قسمت‌های D، E و مجموع بخش‌های D و E افزایش میانگین نمرات به ترتیب 07/3، 57/2 و 82/2 حاصل شد که تمامی این مقادیر معنی‌دار بود. نتیجه‌گیری: اين مطالعه نشان داد انجام تمرین مقاومتی نشستن به ایستادن با وزنه باعث افزایش میزان عملکرد حرکتی درشت بخش D، بخش E و مجموع بخش‌های D و E می‌شود. در ضمن انجام این تمرینات باعث افزایش قدرت ایزومتریک گروه‌های عضلانی اکستانسور ران و اکستانسور زانو می‌شود. اما در میزان قدرت عضلانی گروه‌های پلانتار فلکسور مچ پا تأثیر معنی‌داری مشاهده نشد.

Title: تأثیر بازتوانی قلبی بر کیفیت زندگی بیماران مبتلا به بیماری‌های قلبی- عروقی

http://jrrs.mui.ac.ir/index.php/jrrs/article/view/213

مقدمه: کیفیت زندگی به عنوان یک شاخص مهم در بررسی میزان تأثیر مداخلات در بیماری‌های قلبی- عروقی است. بهبود کیفیت زندگی یکی از اهداف مهم بیمار برای شرکت در برنامه بازتوانی قلبی می‌باشد. در ایران در زمینه تأثیر بازتوانی قلبی بر کیفیت زندگی بیماران قلبی مطالعات کافی صورت نگرفته است. هدف این مطالعه، بررسی تأثیر بازتوانی قلبی بر کیفیت زندگی بیماران قلبی- عروقی بود. مواد و روش‌ها: در این مطالعه نیمه تجربی گذشته‌نگر، پرونده 100 نفر از بیماران قلبی مراجعه‌کننده به مرکز تحقیقات قلب و عروق اصفهان به روش تصادفي ساده بررسي شد. اطلاعات دموگرافيك شامل اندازه قد، وزن و محيط شكم افراد مورد بررسي قرار گرفت. جهت تعيين ريسك بیماران و تدوین برنامه ورزشی قبل از شروع بازتوانی، تست ورزش و اكوكارديوگرافي انجام شد و ظرفیت ورزشی و کسر تخلیه بطن چپ محاسبه گردید. شدت ورزش بیماران بر اساس 60 تا 80 درصد حداکثر ضربان قلب به دست آمده در تست ورزش تعیین مي‌شد. برای تعیین نمره کیفیت زندگی بیماران، پرسش‌نامه استاندارد شده كيفيت زندگي 36-SF توسط فرد آموزش‌دیده برای بیماران تكميل شد. 24 جلسه برنامه بازتواني، هر جلسه شامل گرم كردن، ورزش هوازي، سرد كردن و آرام‌سازي همراه با ارایه رژيم غذايي و 8 جلسه آموزش بيمار در خصوص كاهش عوامل خطر، تحت نظارت تيم بازتواني انجام ‌گرفت. در پايان دوره بازتواني همه آزمون‌ها، آزمايش‌ها و اندازه‌گيري‌ها تکرار ‌شدند. کلیه اطلاعات از پرونده بیماران استخراج گردید و بر اساس گروه سنی (زیر 65 سال و 65 سال به بالا) و جنسیت توسط نرم‌افزار 12SPSS و با آزمون Independent t-test آناليز شد. همچنین برای مقایسه نمره کیفیت زندگی بیماران قبل و بعد از بازتوانی از آزمون Paired t-test استفاده ‌شد. یافته‌ها: پس از بازتوانی قلبی، کیفیت زندگی بیماران از نظر بعد فیزیکی شامل عملکرد فیزیکی (002/0 = P)، محدودیت وظایف به علت مشکلات فیزیکی (001/0 > P)، درد (01/0 = P) و نیز سرزندگی (001/0 = P)، بهبود معنی‌داری داشت. تفاوت معنی‌داری در کیفیت زندگی افراد بالای 65 سال و زیر 65 سال مشاهده نشد (05/0 = P)، ولی کیفیت زندگی زنان نسبت به مردان از نظر عملکرد فیزیکی (03/0 = P) و سلامت روحی (02/0 = P) بهبود بیشتری داشت و در سایر موارد تفاوت معنی‌دار نبود (05/0 = P). نتیجه گیری: نتایج این مطالعه نشان داد که بازتوانی قلبی جامع موجب بهبود کیفیت زندگی بیماران قلبی به ویژه در زنان می‌شود و افراد مسن به اندازه سایر افراد از بازتوانی قلبی سود می‌برند.

Title: بررسی مقايسه‌اي درمان فشرده دو دستی دست- بازو و رويكرد درماني رايج بر کارکرد یک دستی و دو دستی کودکان فلج مغزی نیمه بدن

http://jrrs.mui.ac.ir/index.php/jrrs/article/view/267

مقدمه: بررسي روش حرکت‌درماني با محدوديت اجباري آشکار ساخت که اگر چه اين روش در بهبود کارکرد یک دستی کودکان با فلج مغزي نيمه بدن مؤثر است، اما با محدوديت‌هايي نيز مواجه است. بر اين اساس روش جديدي معرفي و «درمان فشرده دو دستي دست- بازو» نام نهاده شد. هدف این مطالعه، بررسی تأثیر این روش بر کارکرد دو دستی و یک دستی کودکان با فلج نیمه بدن بوده است. مواد و روش‌ها: این پژوهش با طرح کارآزمایی کنترل شده تصادفی یک سو کور بر روی 28 شرکت کننده فلج مغزي نيمه بدن که بنا بر ملاک‌های ورود و خروج در دو گروه درمان فشرده دو دستی دست- بازو و گروه کنترل قرار گرفتند، انجام شد. مداخله در گروه درمان فشرده دو دستی دست- بازو به صورت 6 ساعت، هر روزه در طی 10 روز متوالی انجام شد. در حالی که گروه ديگر از درمان رایج کاردرمانی استفاده می‌کردند. داده‌ها با استفاده از ابزارهای به کار رفته در این پژوهش، آزمون کفايت حرکتي Bruininks–Oseretsky، آزمون کارکرد دستي Jebsen-Taylor، مقیاس بررسی استفاده کارکردی توسط مراقب، جمع‌آوری و با استفاده از روش‌های آماری Independent t-test و Paired t-test تحلیل شد. یافته‌ها: بررسی داده‌های حاصل نشان داد که شرکت کنندگان در گروه حرکت‌درمانی با محدودیت اجباری در متغیرهای چیره دستی، هماهنگی دو طرفه، هماهنگی اندام فوقانی، فعالیت‌های دو دستی، فعالیت‌های یک دستی، ادراک والدین (فراوانی و کیفیت) عملکرد بهتری را نسبت به شرکت کنندگان گروه درمان رایج نشان دادند (05/0 > P)، در حالی كه در خصوص آزمون Jebsen-Taylor تفاوت معنی‌داری بین دو گروه مشاهده نشد. همچنین در بین متغییرهای فوق از پیش‌آزمون تا پس‌آزمون در گروه درمان فشرده دو دستی دست- بازو پیشرفت ملاحظه شد، اما در گروه درمان رایج تفاوت معنی‌داری مشاهده نشد. نتیجه گیری: بر اساس یافته‌های پژوهش، روش درمان فشرده دو دستی دست- بازو هم بر کارکرد یک دستی و هم بر کارکرد دو دستی کودکان با فلج نیمه بدن مؤثر بوده است.

Title: بررسي رابطه مهارت‌هاي اجتماعي و ويژگي‌هاي شخصيتی دانش‌آموزان عادي و داراي لكنت مدارس ابتدایی شهر اصفهان و مقایسه این دو متغیر در دو گروه

http://jrrs.mui.ac.ir/index.php/jrrs/article/view/248

مقدمه: پژوهش حاضر با هدف، بررسي رابطه مهارت‌هاي اجتماعي و وي‍ژگي‌هاي شخصيت دانش‌آموزان عادي و داراي لكنت زبان مقاطع چهارم و پنجم ابتدایی شهر اصفهان و مقایسه آن‌ها در دو گروه صورت گرفت. مواد و روش‌ها: به منظور انجام این مطالعه توصیفی- تحلیلی،30 نفر از دانش‌آموزان داراي لكنت با روش نمونه‌گيري خوشه‌اي چند مرحله‌اي و 30 نفر از دانش‌آموزان به روش نمونه‌گيري تصادفي ساده انتخاب شدند. براي جمع‌آوري داده‌ها از چک‌لیست مهارت‌هاي اجتماعي و پرسش‌نامه شخصيت كودك استفاده شد. داده‌هاي به دست آمده با روش One-way ANOVA و ضريب همبستگي Pearson مورد تجزيه و تحليل قرار گرفت. یافته‌ها: از بين شش عامل شخصيت كودكان فقط بين عامل عزت نفس در بين دو گروه تفاوت معنی‌دار وجود داشت. همچنين نتايج ضرایب همبستگی نشان داد كه در گروه مبتلا به لکنت، بين كنترل عواطف و عامل وابستگي رابطه منفی و بين كنترل عواطف و عامل‌های عزت نفس و خودكار‌امدي رابطه مثبت و در گروه دانش‌آموزان عادی بین دیدگاه منفی به جهان و رفتار در گروه رابطه منفی وجود دارد. نتیجه گیری: با توجه به داده‌هاي به دست آمده، مي‌توان نتيجه گرفت که به دلیل تفاوت در توانش عملی کاربرد زبان بین دو گروه عادی و مبتلا به لکنت و در نتیجه برآورد منفی از توانایی‌های خود در گروه مبتلا به لکنت، این گروه از عزت نفس پایین‌تری نسبت به گروه عادی برخوردارند. از طرف دیگر در گروه مبتلا به لکنت، کنترل عواطف که از فرصت‌های اجتماعی بالا ناشی می‌شود، عدم وابستگی، عزت نفس و خودکارامدی را می‌تواند به همراه داشته باشد و بالعکس. در گروه دانش‌آموزان عادی ادراک منفی از اجتماع که می‌تواند خواستگاه‌های مختلفی داشته باشد، پیش‌بینی کننده روابط بین فردی غیر مؤثر می‌باشد.

Title: بررسی ویژگی‌های آکوستیکی صوتی کودکان دارای بی‌کفایتی دریچه کامی- حلقی 4 تا 8 ساله و مقایسه آن با هم‌سالان طبیعی

http://jrrs.mui.ac.ir/index.php/jrrs/article/view/316

مقدمه: به کارگیری بعضی از مکانیزم‌های جبرانی برای سازگاری با بی‌کفایتی دریچه کامی- حلقی (Velopharyngeal insufficiency یا VPI) جهت کاهش پرخیشومی و افزایش بلندی صدا، فشار بیشتری را بر حنجره وارد می‌کند و در نتیجه می‌تواند منجر به اختلالات صوتی گردد. تعدادی از مطالعات به ویژگی‌های صوتی افراد با VPI پرداخته‌اند. تصویری که از این تحقیقات به وجود می‌آید، نشان دهنده یافته‌های متنوع و حتی متضاد می‌باشد. این مطالعه به منظور اندازه‌گیری دقیقی از ویژگی‌های آکوستیکی صوتی شامل فرکانس پایه، جیتر (Jitter) و شیمر (Shimmer) در کودکان با VPI 4 تا 8 ساله و کودکان طبیعی هم سن آن‌ها و پس از آن مقایسه هر یک از این ویژگی‌ها صورت گرفت. مواد و روش‌ها: این مطالعه توصیفی- تحلیلی و مقطعی بود که بر روی 80 کودک، 48 کودک در گروه کنترل و 32 کودک دارای VPI صورت گرفت. کودکان بر اساس آزمون پارامتر جهانی مورد ارزیابی قرار گرفتند و نمونه‌هایی که دارای پرخیشومی متوسط و شدید بودند، واکه /a/ را برای حداقل 4 ثانیه کشیدند و سپس فرکانس پایه، جیتر و شیمر صداهای ضبط شده به وسیله نرم‌افزار Dr Speech مورد تجزیه و تحلیل قرار گرفت. داده‌های به دست آمده با استفاده از روش‌های آمار توصیفی و تحلیلی (میانگین، انحراف معیار و آزمون t) بر اساس اهداف پژوهش مورد تجزیه و تحلیل قرار گرفت. یافته‌ها: تفاوت معنی‌داری بین میانگین فرکانس پایه، جیتر و شیمر در دو گروه کودکان دارای VPI و طبیعی 4 تا 8 ساله وجود نداشت. نتیجه‎گیری: با توجه به یافته‌های این تحقیق می‌توان نتیجه گرفت که در کودکان دارای VPI در محدوده سنی 4 تا 8 سال، VPI تأثیر چندانی بر فعالیت‌های حنجره‌ای نمی‌گذارد. شاید به این علت که در این سن هنوز راهکارهای سازگاری با VPI منجر به تغییرات بارز در ویژگی‌های آکوستیکی تارهای صوتی نمی‌شوند.

Title: بررسي پايايي نسخه فارسي مقياس سنجش تأثيرات سكته مغزي در افراد مبتلا به سكته مغزي

http://jrrs.mui.ac.ir/index.php/jrrs/article/view/348

مقدمه: هدف از پژوهش حاضر فراهم‌‌سازي ابزاري پايا به زبان فارسي جهت سنجش تأثيرات سكته مغزي در افراد مبتلا به سكته مغزي، از طريق ترجمه و معادل‌سازي پرسش‌نامه «مقياس سنجش تأثيرات سكته مغزي» به زبان فارسي و بررسي پايايي نسخه فارسي معادل‌سازي شده در این افراد بود. مواد و روش‌ها: پرسش‌نامه «مقياس سنجش تأثيرات سكته مغزي» طي سه مرحله طبق روش IQOLA (International quality of life assessment project) به زبان فارسي ترجمه و معادل‌سازي شد. براي بررسي تكرارپذيري، پرسش‌نامه در دو نوبت با فاصله حداكثر 3 هفته در اختيار 10 نفر فارسي‌زبان مبتلا به سکته مغزی قرار گرفت و برای بررسی هم‌خوانی درونی در اختیار 60 نفر كه طبق تشخيص پزشك مبتلا به سكته مغزي بودند و جهت دريافت خدمات پزشكي و توان‌بخشي به دو بيمارستان كاشاني و الزهراء‌(س) در شهر اصفهان طی سال‌های 86-1384 مراجعه كرده بودند، قرار گرفت. روش نمونه‌گيري به صورت ساده و غير احتمالي بود. داده‌هاي به دست آمده از لحاظ تكرارپذيري در دفعات آزمون و هم‌خواني دروني گزینه‌ها، تحت تجزيه و تحليل آماري قرار گرفت. یافته‌ها: ضرايب Cronbach's alpha که برای بررسی هم‌خواني دروني حوزه‌هاي پرسش‌نامه «مقياس سنجش تأثيرات سكته مغزي» به کار رفته بود، ما بین 69/0 تا 87/0به دست آمد كه نشان مي‌دهد حوزه‌های متفاوت مقياس سنجش تأثيرات سكته مغزي (Stroke impact scale یا SIS) يك مفهوم را ارزيابي مي‌كنند. ضريب ICC (Intraclass correlation coefficient) كه براي بررسي تكرارپذيري نسبي نسخه فارسي پرسش‌نامه «مقياس سنجش تأثيرات سكته مغزي» در دفعات آزمون به كار رفته بود، محاسبه شد و بالاتر از 78/0 به دست آمد كه از مقدار قابل قبول 70/0 بالاتر بود. این مقدار تكرارپذيري خوب نسخه فارسي پرسش‌نامه «مقياس سنجش تأثيرات سكته مغزي» را نشان مي‌داد. همچنین ضرایب همبستگی دو بار تکرار آزمون از 71/0 بالاتر بود که این همبستگی قوی و بالا نشان می‌داد، با گذشت زمان این آزمون قابلیت اندازه‌گیری مفهوم مورد ارزیابی را دارد. نتیجه گیری: اين يافته‌ها نشان داد كه پرسش‌نامه «مقياس سنجش تأثيرات سكته مغزي» از پایایی خوبی برخوردار است و مي‌توان آن را به عنوان ابزاری پایا به منظور سنجش تأثیرات بیماری در افراد مبتلا به سکته مغزی به کار برد.

Title: بررسی فراوانی بیماران مبتلا به پرتس مراجعه کننده به مرکز ارتوپدی هلال احمر اصفهان

http://jrrs.mui.ac.ir/index.php/jrrs/article/view/270

مقدمه: بیماری پرتس یکی از بیماری‌های شایع کودکان است. در این بیماری سر استخوان ران به علت اختلال در خون‌رسانی دچار نکروز شده است. این استئونکروزیس دست‌خوش تغییراتی می‌شود و بافت جدید جایگزین بافت از بین رفته می‌گردد و شکل استخوان تغییر می‌کند. اين بيماري در دهه اول زندگي بروز مي‌كند. با توجه به يافت نشدن پژوهشي در ارتباط با شيوع و فراواني پرتس در ايران، اين مطالعه با هدف بررسي فراواني و علل ايجاد اين بيماري انجام شد. مواد و روش‌ها: این مطالعه توصیفی گذشته‌نگر بر روی مراجعین به مرکز درمانی هلال احمر اصفهان انجام شد. اطلاعات مربوط به ارزيابي و معاينه پزشك متخصص و بيوگرافي و اندازه‌گيري‌هاي لازم مثل ميزان كوتاهي كه توسط تخته كوتاهي انجام شده بود، در دو پرسش‌نامه استخراج گرديد. این افراد هیچ گونه سابقه شکستگی ناحیه هیپ و آسیب تروماتیک مفصل هیپ (دررفتگی و نیمه دررفتگی) نداشتند. در اين مطالعه از آزمون‌هاي شاخص مركزي و پراكندگي و همبستگي و 2χ استفاده گردید. یافته‌ها: از 77 نفر بيمار مراجعه كننده، نسبت پسر به دختر 4 به 1 بود و سن ابتلا در اين بيماران از يك سال تا سيزده سال متغيير بود (ميانگين 7 سال). 61 درصد درد و لنگش همزمان، 29 درصد لنگش و 10 درصد با درد مراجعه كرده بودند. ميانگين كوتاهی 6/1 سانتي‌متر بود. سمت راست و چپ يكسان و هر دو پا 11 درصد درگير بود. نسبت زندگي شهري به روستایي بيشتر بود. تنها 5 درصد از اين بيماران داراي سابقه فاميلي بودند. ميانگين تعداد فرزندان 3 فرزند بود. نتیجه گیری: بر اساس نتايج حاصله از مطالعه حاضر، ميزان فراواني بيماري پرتس در جامعه مورد مطالعه با در نظر گرفتن جنسيت، 82 درصد پسر و 18 درصد دختر بود. به علاوه در این افراد ميزان درد و لنگش 61 درصد و ميزان كوتاهي 6/1 سانتي‌متر بود. در مورد سمت ضايعه در 44، 43 و 11 درصد افراد به ترتيب در سمت چپ، راست و هر دو طرف ضایعه وجود داشت. نتايج فوق با اكثر مطالعات مشابه هم‌خواني داشت. اين مطالعه نشان داد که رابطه معني‌داري بين زندگي شهري و ميزان ابتلا به پرتس وجود دارد (05/0 > P).

Title: بررسی و مقایسه اثر پیچیدگی نحوی بر میزان ناروانی گفتار بزرگ‌سالان لکنتی و غیرلکنتی فارسی‌زبان در تکلیف تقلید جملات

http://jrrs.mui.ac.ir/index.php/jrrs/article/view/223

مقدمه: چگونگی ارتباط میان پیچیدگی نحوی با ناروانی‌های لکنت در سال‌های اخیر مورد توجه محققین قرار گرفته است. هدف از این پژوهش، بررسی تأثیر پیچیدگی نحوی بر میزان ناروانی گفتار بزرگ‌سالان لکنتی و غیرلکنتی فارسی‌زبان بود. نتایج به‌ دست آمده می‌تواند جهت شناخت بیشتر ماهیت لکنت بزرگ‌سال و نیز یافتن شیوه‌های مناسب‌تر برای درمان آن کاربرد داشته باشد. مواد و روش‌ها: در این مطالعه‌ توصیفی- تحلیلی از نوع مقطعی، دو گروه شامل 15 بزرگ‌سال لکنتی و 15 بزرگ‌سال غیرلکنتی تک‌زبانه‌ و فارسی‌زبان بین سنین 15 تا 50 سال مورد بررسی قرار گرفتند؛ به طوری که دو گروه مورد مطالعه از لحاظ سن و جنس با یکدیگر مطابقت داشتند. برای بررسی ناروانی، دو دسته جملات شامل ساده و پیچیده برای هر گروه در نظر‌گرفته شد که به ترتیب بر طول جملات هر دسته یک تک‌واژ افزوده شد. پس از اجرای تکلیف تقلید جملات در دوگروه، اطلاعات به ‌دست آمده توسط نرم‌افزار SPSS و با استفاده از آزمون آماری Paired t-test مورد تجزیه ‌و‌ تحلیل قرار گرفت. یافته‌ها: با افزایش پیچیدگی نحوی، میزان ناروانی گفتار بزرگ‌سالان لکنتی و غیرلکنتی، افزایش معنی‌داری نداشت (05/0 > P). نتیجه گیری: بر اساس نتایج با افزایش پیچیدگی نحوی، میزان ناروانی گفتار بزرگ‌سالان لکنتی و غیرلکنتی فارسی‌زبان در سطح تقلید جملات، افزایش معنی‌داری نداشته است. با توجه به یافته‌های این مطالعه، به نظر می‌رسد که توجه به عوامل زبانی در کودکان و بزرگ‌سالان لکنتی ضروری بوده و نیز توجه به افزایش تدریجی پیچیدگی نحوی در درمان از پیشنهادات مفید می‌باشد.

Title: تأثیر یک دوره تمرینات پیلاتس بر استئوآرتریت زانوی مردان ورزشکار سالمند

http://jrrs.mui.ac.ir/index.php/jrrs/article/view/211

مقدمه: امروزه شرکت در فعاليت بدني در پيش‌گيري از بيماري‌هاي مزمني همچون استئوآرتريت پيشنهاد مي‌شود. هدف پژوهش حاضر، بررسی تأثیر یک دوره تمرینات پیلاتس بر شدت درد زانو، ميزان علایم و مشكلات عملكرد حركتي در فعاليت‌هاي روزانه، ورزشي، تفريحي و کيفيت زندگي مردان ورزشکار سالمند بود. مواد و روش‌ها: 15 معلم تربیت بدنی مبتلا به استئوآرتریت زانو با ميانگين و انحراف استاندارد سن 47/6 ± 57 سال، قد 49/8 ± 43/176 سانتي‌متر، وزن 81/8 ± 21/76 کيلوگرم به صورت هدفمند، به عنوان آزمودني انتخاب شدند. برای جمع‌آوری داده‌ها از پرسش‌نامه جهانی و بومی‌سازی شده پیامد صدمات زانو و استئوآرتریت استفاده شد. نمونه‌ها تمرینات پیلاتس را به مدت 8 هفته زیر نظر متخصص پیلاتس انجام دادند. براي تجزيه و تحليل داده‌ها از Correlated t-test در سطح معنی‌داري استفاده گردید (05/0 < P). یافته‌ها: بین پیش تا پس‌آزمون ميانگين شدت درد زانو (144/0 = P) و همچنین میزان علایم (367/0 = P) تفاوت معني‌داري وجود نداشت، در حالی که عملکرد حرکتی در فعالیت‌های روزانه (001/0 = P)، ورزشي، تفريحي (007/0 = P) و کیفیت زندگی (002/0 = P) پس از اجرای پروتکل به میزان معنی‌داری بهبود یافته بود. نتیجه گیری: نتايج نشان داد که تمرينات پیلاتس می‌تواند به عنوان یک روش تمرینی ایمن و مؤثر در بهبود عملکرد و کیفیت زندگی مردان سالمند داراي استئوآرتریت زانو مورد توجه قرار گیرد.

Title: بررسی آهنگ گفتار در جمله‌های خبری زبان فارسی

http://jrrs.mui.ac.ir/index.php/jrrs/article/view/222

مقدمه: نوای گفتار از مؤلفه‌های مهم برقراری ارتباط است که شامل آهنگ، زیروبمی، بلندی، تکیه، وزن، دیرش و ... می‌باشد. آهنگ عبارت است از تغییراتی که در زیروبمی صدا در گفتار پیوسته در سطح جمله رخ می‌دهد. دامنه تغییرات بسامد پایه که در این تحقیق مد نظر قرار گرفته است، از پایین‌ترین تا بالاترین بسامدی که در بیان جمله توسط فرد تولید شده است، را در بر می‌گیرد. هدف این پژوهش بررسی مؤلفه‌هایی از نوا شامل دامنه تغییرات بسامد پایه و منحنی آهنگ در جملات خبری در بزرگ‌سالان طبیعی فارسی زبان بوده است، تا با تکیه بر جنبه‌های آزمایشگاهی ویژگی‌های این بعد از گفتار مشخص شود. مواد و روش‌ها: پژوهش از نوع مقطعی و توصیفی- تحلیلی بود. آزمودنی‌ها شامل 134 زن و مرد 18 تا 30 ساله طبیعی فارسی زبان بودند. در این پژوهش 2 جمله خبری مورد بررسی قرار گرفت. نمونه‌های صوتی با استفاده از برنامهDr.speech  نرم‌افزار Real analysis تجزیه و تحلیل شده، داده‌ها با آزمون آماری آنالیز واریانس یک طرفه مورد بررسی قرار گرفتند و منحنی آهنگ جمله‌ها رسم شد. یافته‌ها: منحنی آهنگ در زنان و مردان در هر دو جمله مشابه است. دامنه تغییرات بسامد پایه در زنان و مردان با هم تفاوت معنی‌دار داشت (001/0 ≤ P). نتیجه گیری: منحنی آهنگ در جمله‌های خبری در پایان حالت افتان داشت. زنان بیش از مردان از تغییرات بسامد پایه در بیان جمله برای انتقال حالت خبری جمله استفاده می‌کردند.

Title: مروری بر درمان‌های فیزیکی مبتلايان به سندرم دردناک مفصل پتلوفمورال

http://jrrs.mui.ac.ir/index.php/jrrs/article/view/283

یکی از شایع‌ترین اختلالات اسکلتی- عضلانی ناحیه زانو در بالغین جوان، سندرم دردناک مفصل پتلوفمورال (Patellofemoral pain syndrome یا PFPS) می‌باشد. تاکنون علت ابتلا به این سندرم به درستی مشخص نشده است. با این وجود، پژوهش‌گران بر این باورند که چندین علت از جمله عوامل مستعد کننده بیومکانیکی، بافت نرم، عدم تعادل ماهیچه‌ای و اختلالات بیومکانیکی ناحیه پا و نواحی پروگزیمال ران برای ابتلا به این سندرم دخیل هستند. با توجه به میزان بالای ابتلا به این سندرم در جوانان بالغ و تنوع دلایل مطرح شده جهت بروز این عارضه، در مقاله حاضر تلاش شده است که ضمن بیان دلایل احتمالی، درمان‌های فیزیکی رایج این عارضه با توجه به دیدگاه‌های مختلف و پژوهش‌های نوین صورت گرفته طی دو دهه اخیر بیان شود. ذکر درمان‌های برتر سندرم دردناک مفصل پتلوفمورال (PFPS)، به درمان‌گران فیزیکی کمک خواهد کرد تا بر اساس علت ضایعه و شرایط موجود هر بیمار به درمان مؤثرتر این عارضه بپردازند. بنابراین پژوهش‌گر حاضر با مراجعه به متون منتشر شده در پایگاه‌های اطلاعاتی Pubmed، Science direct، Web of knowledge و Google scholar نسبت به جمع‌آوری داده‌ها و اعلام نتایج آن‌ها اقدام نموده است.
